# Time-varying boundaries for diffusion models of decision making and response time

**DOI:** 10.3389/fpsyg.2014.01364

**Published:** 2014-12-09

**Authors:** Shunan Zhang, Michael D. Lee, Joachim Vandekerckhove, Gunter Maris, Eric-Jan Wagenmakers

**Affiliations:** ^1^Department of Cognitive Sciences, University of CaliforniaIrvine, Irvine, CA, USA; ^2^Psychological Methods, University of AmsterdamAmsterdam, Netherlands

**Keywords:** accumulator model, collapsing bounds, model equivalence, sequential sampling, first-passage time

## Abstract

Diffusion models are widely-used and successful accounts of the time course of two-choice decision making. Most diffusion models assume constant boundaries, which are the threshold levels of evidence that must be sampled from a stimulus to reach a decision. We summarize theoretical results from statistics that relate distributions of decisions and response times to diffusion models with time-varying boundaries. We then develop a computational method for finding time-varying boundaries from empirical data, and apply our new method to two problems. The first problem involves finding the time-varying boundaries that make diffusion models equivalent to the alternative sequential sampling class of accumulator models. The second problem involves finding the time-varying boundaries, at the individual level, that best fit empirical data for perceptual stimuli that provide equal evidence for both decision alternatives. We discuss the theoretical and modeling implications of using time-varying boundaries in diffusion models, as well as the limitations and potential of our approach to their inference.

## 1. Introduction

Being able to make a timely choice between two alternatives is a cornerstone of human cognition, and a long-standing focus of experimentation and theorizing in cognitive psychology. One widely used approach to modeling the time course of decision making comes from the class of *sequential sampling* models (Link and Heath, 1975; Ratcliff, 1978; Vickers, 1979; Luce, 1986; Busemeyer and Townsend, 1993; Usher and McClelland, 2001; Ratcliff and McKoon, 2008). In these models, people are assumed to gather information, piece by piece, until they have accrued enough evidence in favor of one or other alternative to justify that decision. The most prominent and popular sequential sampling models are *diffusion models*, which make the assumption that the samples of evidence come from a Gaussian distribution, and are accumulated according to a random walk that becomes a diffusion process as the time-step between samples approaches a limit of zero (Ratcliff, 1980, 1985, 1988, 2013; Ratcliff and Rouder, 1998, 2000; Zandt and McKoon, 1999).

**Figure 1:**
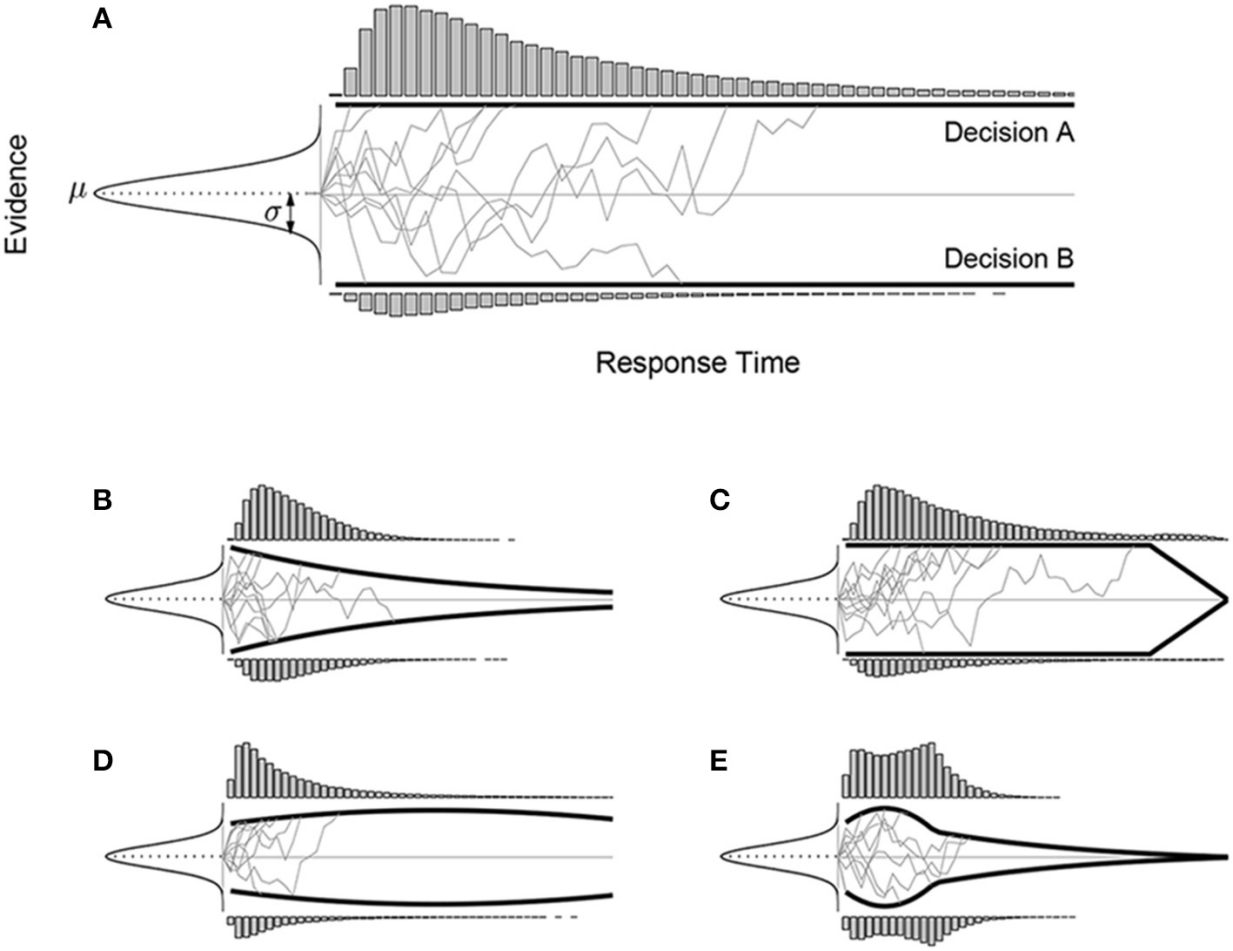
**The basic drift diffusion sequential sampling model of two-choice decision-making and response times (A), and variants involving time-varying boundaries (B–E)**. In each case, evidence values are sampled from the same Gaussian distribution with mean μ and standard deviation σ, but different boundaries lead to different response time distributions for the two alternative decisions.

The basic diffusion model assumptions and operation are shown graphically in Figure 1A. Evidence values are sampled from a Gaussian with mean μ and standard deviation σ. These values are accumulated in a single tally until the tally reaches either the upper or lower boundaries shown by solid black lines. Once the tally reaches a boundary, evidence accumulation stops, and the model makes the decision associated with the boundary that was reached, with a response time corresponding to the number of samples taken. Figure 1A shows 10 example tallies by thin gray lines. It also shows by histograms at the boundaries the distribution of response times for each decision.

When applied to account for human decision-making, diffusion models are usually extended beyond the basic form shown in Figure 1A. Most often, additional parameters are added, introducing variability to the evidence accrual process, or incorporating encoding and retrieval processes, or processes that cause leakage or drift in the tallies (e.g., Ratcliff, 1978; Busemeyer and Townsend, 1992; Usher and McClelland, 2001; Ratcliff and McKoon, 2008). In these expanded forms, diffusion models have been widely applied to model human decision-making for a variety of tasks, including: many simple perceptual decisions like coherent motion detection, line length comparison, and brightness discrimination (e.g., Ratcliff and Rouder, 1998; Ratcliff et al., 2003); simple cognitive tasks, like lexical decision (e.g., Ratcliff et al., 2004a; Wagenmakers et al., 2008); basic information processing tasks like choice reaction time (e.g., Laming, 1968; Link and Heath, 1975); memory processes (e.g., Ratcliff et al., 2004b; White et al., 2009); and a range of more complex cognitive decision tasks, including categorization and classification (e.g., Nosofsky and Palmeri, 1997), heuristic decision-making (e.g., Lee and Cummins, 2004; Lee and Zhang, 2012), and judgment and choice (e.g., Wallsten and Barton, 1982; Busemeyer and Rapoport, 1988; Busemeyer and Townsend, 1993; Diederich, 1997).

One area that has been under-explored in diffusion modeling involves the use of time-varying boundaries. The vast majority of diffusion models in psychology use constant boundaries, as shown in Figure 1A. Constant boundaries were originally motivated by optimality properties, in the sense that setting a boundary corresponds to setting a Type I error rate, as in the sequential probability ratio test (Wald and Wolfowitz, 1948). Some previous diffusion models, however, have considered within-trial changes in boundaries, usually in the form of that converge over time (e.g., Pickett, 1968; Rapoport and Burkheimer, 1971; Clay and Goel, 1973; Viviani, 1979; Hockley and Murdock, 1987; Busemeyer and Rapoport, 1988; Heath, 1992; Frazier and Yu, 2008; Milosavljevic et al., 2010). Considering time-varying boundaries has become an active area of research recently, both in the context of models that combine neuro-psychological data with formal modeling of decision processes (e.g., Cisek et al., 2009; Gluth et al., 2012; Ratcliff and Frank, 2012; Thura et al., 2012), and in the context of studying the theoretical relationships between, and the falsifiability of, sequential-sampling models (Jones and Dzhafarov, 2014).

Figures 1B–E show examples of different time-varying boundaries, and the distributions of decisions and response times they produce for the same Gaussian evidence distribution. It is clear that allowing this flexibility in diffusion models makes them capable of capturing both qualitatively and quantitatively different decision and response time patterns. One reason for wanting this flexibility is to accommodate patterns seen in empirical data, especially arising from experimental task demands. Time-varying boundaries could be regarded, for example, as implementing time pressure, urgency-gating, or deadlines within a single decision trial (Ditterich, 2006; Frazier and Yu, 2008; Cisek et al., 2009). Another reason for considering time-varying boundaries is to broaden the types of optimality in decision-making that can be considered by diffusion models (e.g., Drugowitsch et al., 2012; Ratcliff and Frank, 2012). While constant boundaries, as noted above, optimize single decisions with respect to a fixed Type I error rate, this is not the only possible criterion decision makers might optimize. For example, in some situations—such as when there is not fixed number of decisions to be made, but rather a fixed length of time in which any number of decisions can be made—it might be more important to optimize the *rate* at which correct decisions are made, rather than focus on the correctness of each individual trial. A specific example is provided by Drugowitsch et al. (2012, Figure 3C), who showed that the optimal boundaries for the Wiener diffusion model are decreasing when there are multiple levels of difficulty and intermixed trials in a 2-alternative-forced-choice (2AFC) task^1^. It is when there is only one level of difficulty in the task that the SPRT Optimality Theorem guarantees that the Wiener process with constant boundaries (among all possible models) maximize any reward criteria that are monotonically non-increasing with respect to the response time (e.g., Bogacz et al., 2006). Many real-world decision-making situations are more general, and so afford possibility that time-varying boundaries may be optimal. In general, different time-varying boundaries can often be interpreted as optimizing different sorts of criteria relevant to different decision-making situations.

In this paper, we develop a computational method for finding time-varying boundaries from response time distributions that does not constrain their form and does not commit to specific theoretical assumptions about optimality. Our method is motivated by relevant results from statistics that relate pattern of decisions and response times to diffusion models with time-varying boundaries. Our method does not constrain the time-varying boundaries to a parametric family, but does require knowing the mean and standard deviation of the Gaussian evidence distribution.

To demonstrate our method, we apply it to two concrete problems. The first problem involves equating diffusion models with an alternative class of sequential sampling models, known as accumulator models, and requires applying our method to simulated data. The second problem involves finding the time-varying boundaries in a perceptual decision-making task in the case where the visual stimulus provides the same level of evidence in favor of either decision alternative. Applying our method at the individual level, this second application allows us to consider basic individual differences in the thresholds people use to make a simple perceptual decision. We conclude with a discussion of the theoretical and modeling implications of using time-varying boundaries for diffusion models, as well as considering the limitations and potential of our method.

## 2. Finding time-varying boundaries

We approach the problem of finding time-varying boundaries as one of solving an inverse problem numerically. There are three important elements to our approach. The first element is having a method for generating the decision and response time distributions that are produced by a known Gaussian evidence distribution and known time-varying boundaries. The second element is a theoretical result that guarantees that any decision and response time distribution, for a given Gaussian evidence distribution, is generated by unique time-varying boundaries. The third element is a numerical method for finding those boundaries, given the Gaussian evidence distribution and decision and response time distribution. In this section, we present each of these three elements in turn.

### 2.1. Generating data from diffusion models with time-varying boundaries

We study a diffusion model sampling evidence from the Gaussian distribution with constant mean μ and standard deviation σ, but with the additional flexibility of having time-varying boundaries. This model generates a decision probability *p*^diff^ and response time distributions *r*^diff^_*A*_ and *r*^diff^_*B*_ for the two decisions. Denoting the decision boundaries as *a_A_* and *a_B_* for the two decisions, where *a_A_* and *a_B_* are both time-dependent functions, the diffusion model can be conceived as a mapping

(1) $$m^{\text{diff}}:(\mu ,\sigma ,a_{A},a_{B})\to (p^{\text{diff}},r_{A}^{\text{diff}},r_{B}^{\text{diff}}).$$

The mapping *m*^diff^ has been studied in the statistics literature, and an effective approach using the analysis of renewal equations has been developed (Durbin, 1971; Buonocore et al., 1987, 1990). Buonocore et al. (1990) provide an efficient algorithm to compute the response time distributions for time-varying boundaries. A summary of these methods well-suited for psychologists is given by Smith (2000). In particular, data can be generated from a diffusion model with flexible boundaries using general Markov process methods. Because (Smith, 2000) does not provide results for exactly the diffusion model we use (we use a special case of a more general one that is provided), we give explicitly the details needed to reproduce our results.

The basic idea is to specify how sample evidence paths *X*(*t*) are generated, and then use existing results that give the first passage time distributions through arbitrary boundaries that are continuously differentiable. The diffusion model we study corresponds to a Wiener process with a constant drift ξ and infinitesimal variance *s*^2^.^2^ Specifying the sample paths for this process is done by specifying the transition density

(2) $$\begin{array}{l} f\left(x,t|y,\tau \right)=\frac{\text{d}}{\text{d}x}F\left(x,t|y,\tau \right)=\frac{1}{\sqrt{2\pi s^{2}\left(t-\tau \right)}}\exp\\ \;\left(-\frac{{\left(x-y-\xi \left(t-\tau \right)\right)}^{2}}{2s^{2}\left(t-\tau \right)}\right) \end{array}$$

where *F*(*x*, *t* | *y*, τ) is the probability of the tally being less than or equal to *x* at time *t*, given its value at an earlier time τ was *y*. Notice that both *f* and *F* are the densities when there is no boundary.

The first passage time densities through the time-varying absorbing boundaries, *a*_A_ and *a*_B_, are denoted by *g_A_*(*a_A_*(*t*), *t* | *x*_0_, *t*_0_) and *g_B_*(*a_B_*(*t*), *t* | *x*_0_, *t*_0_), where *x*_0_ and *t*_0_ are the initial state and time. Analysis using the renewal equation (e.g., Durbin, 1971) yields the *Volterra equations* of the relationship between the transition density and the first passage time densities (Smith, 2000, Equation 41):

(3) $$\begin{array}{l} f(a_{A}(t),t|x_{0},t_{0})=\int_{t_{0}}^{t}g_{A}(a_{A}(\tau ),\tau |x_{0},t_{0})f(a_{A}(t),t|a_{A}(\tau ),\tau )d\tau \\ \;+\text{​}\int_{t_{0}}^{t}\text{​}g_{B}(a_{B}(\tau ),\tau |x_{0},t_{0})f(a_{A}(t),t|a_{B}(\tau ),\tau )d\tau \\ f(a_{B}(t),t|x_{0},t_{0})=\int_{t_{0}}^{t}g_{B}(a_{B}(\tau ),\tau |x_{0},t_{0})f(a_{B}(t),t|a_{B}(\tau ),\tau )d\tau \\ \;+\text{​}\int_{t_{0}}^{t}g_{A}(a_{A}(\tau ),\tau |x_{0},t_{0})f(a_{B}(t),t|a_{A}(\tau ),\tau )d\tau \text{​​​​​​​​} \end{array}$$

In principle, these equations are soluble, but *f*(*x*, *t* | *y*, τ) is singular as *t* approaches τ, therefore Equation 3 needs to be transformed stably for practical approximation methods. A detailed description of the equation and the singularity issue can be found in Smith (2000, pp. 430–432). The kernels of the transformed equations can be found using the method developed by Buonocore et al. (1987, 1990) and detailed by Smith, (2000, pp. 441–446). By letting μ(*s*) = μ = constant in Equation 57 of Smith (2000), the proper function is

(4) $$\Psi \left(a\left(t\right),t|y,\tau \right)=\frac{f\left(a\left(t\right),t|y,\tau \right)}{2}\left(a^{\prime }\left(t\right)-\frac{a\left(t\right)-y}{t-\tau }\right)$$

where *a*(*t*) takes the form of *a_A_* or *a_B_*, and *a*′(*t*) denotes the first derivative of the boundary. With these results in place, diffusion model data can be produced directly from the first passage time densities, *g_A_* and *g_B_*, which are the same as *g*_1_ and *g*_2_ in Equations 47a and 47b of Smith (2000).

### 2.2. Theoretical results for the inverse problem

The inverse first passage time problem—finding the boundaries, given the evidence distribution and decision and response time distribution—is much harder than the first passage time problem. It has, however, been studied in the fields of applied mathematics and statistics (e.g., Capocelli and Ricciardi, 1972; Cheng et al., 2006; Chen et al., 2011).

Analytic expressions for the boundaries are rarely available and previous research has usually focused on developing numerical methods for computing the boundary. Theoretical work has been relatively scarce. Early work by Capocelli and Ricciardi (1972) addressed the problem of under what conditions an arbitrary density function can be interpreted as the first passage density function for a continuous one-dimensional Markov process with constant boundaries and a known starting value. Some relevant results, in the context of the types of sequential sampling models used to model human decision-making, were obtained. In particular, Capocelli and Ricciardi, (1972, corollary 2.2) found the technical conditions that guarantee the uniqueness of the solution, if it exists, for the Wiener-Lévy and the Ornstein-Uhlenbeck diffusion processes with specified initial condition.

Cheng et al. (2006) were the first to study the well-posedness—that is, the existence and uniqueness—of a specific inverse first-passage time problem close to that of interest in our study. Cheng et al. (2006) addressed the case where a diffusion model has a single boundary, so that there is only one possible decision, and the response time for that decision is being measured. For that case, they proved that for any probability density function *q*, there exists a unique *viscosity* solution to the inverse-first-passage-time problem (i.e., a unique boundary exists under weak assumptions of differentiability). Analogous results for the two-boundary case of direct interest remain an open (and active) research question in the statistics literature. To date, there is no proof that the numerical method developed in the next section of the paper always finds a unique solution.

### 2.3. A numerical method for finding time-varying boundaries

Zucca and Sacerdote (2009) and Song and Zipkin (2011) developed numerical methods for finding time-varying boundaries in the one-boundary case. Because we are interested in diffusion models with two time-varying boundaries, we rely on the approach used by Buonocore et al. (1990). In essence, our method applies this approach, previously used as a forward method only, to the problem of finding two time-varying boundaries.

Algorithm 1 presents the main part of our numerical method for computing the time-varying boundaries as pseudo code. The aim of the algorithm is to find the two boundaries such that the first passage time densities of the process through those boundaries are equal to two desired specific density functions. The algorithm sets the interval between sampling steps to be a small value λ, and calculates the probabilities *P_A,n_* and *P_B,n_* that decision alternatives “A” and “B,” respectively, will be chosen after *n* samples. In practice, *P_A,n_* and *P_B,n_* can be obtained by discretizing the empirical RT distributions for the two alternatives. For the diffusion model discretized to the same sampling interval λ, and using the same Gaussian evidence distribution, the drift rate is ξ = μ/λ and the diffusion coefficient is *s*, where *s*^2^ = σ^2^/λ. The first-order derivative of the boundary at step *n* can be approximated by *a*′ (*n*) = [*a* (*n*) − *a* (*n* − 1)]/λ. These values allow the calculation of Equations 2 and 4 above.

**Algorithm 1:** **Compute the discretized boundaries *a_A_* (*n*) and *a_B_* (*n*), *n* = 1, 2, …, with input μ, σ, *P_A_*, *n*, and *P_B_*, *n***.

Discretize [0, 1] into I small intervals (grid for the boundary)
**for** *n* = 1 to *N* **do**
Compute *P_A,n_* and *P_B,n_*
**for** *i* = 1 to *I* **do**
*c_A_* (*i*) = *i*/*I*
*c_B_* (*i*) = −*i*/*I*
*q_A_*(*i*) ← *g_A_* (*c_A_* (*i*), *n*λ | *x*_0_ = 0, *t*_0_ = 0)
*q_B_* (*i*) ← *g_B_* (*c_B_* (*i*), *n*λ | *x_0_* = 0, *t*_0_ = 0) *g_A_*, *g_B_* as in
Smith (2000), Equation 47
**end for**
*a_A_* (*n*) ← arg min_*i*_ | *q_A_* (*i*)λ − *P_A,n_* | / *I*
*a_B_* (*n*) ← − arg min_*i*_ | *q_B_* (*i*)λ − *P_B,n_* | / *I*
**end for**

The algorithm finds the time-varying boundary through a point-wise approach to its construction, receiving samples from the same Gaussian evidence distribution with mean μ and standard deviation σ. Because the boundaries scale with σ without changing shape, and our assumption that the decision process starts without bias, the initial values of the boundaries can be fixed at +1 and −1, without loss of generality.

The algorithm now sets the equalities *g_A_* (2)λ = *P*_*A*, 2_ and *g_B_* (2)λ = *P*_*B*, 2_, allowing for the solution of the boundaries at the second sample *a_A_* (2) and *a_B_* (2). These steps of the algorithm are now repeated for all of the samples, to find both boundaries in their entirety. Once *a_A_* (1),… *a_A_* (*n*), and *a_B_* (1),… *a_B_* (*n*) are available, it is possible to solve for *a_A_* (*n* + 1) and *a_B_* (*n* + 1) by setting the first passage time densities to be equal, so that *g_A_* (*n* + 1)λ = *P*_*A,n* + 1_ and *g_B_* (*n* + 1)λ = *P*_*B,n* + 1_.

Our algorithm solves the equations at each sample using a simple grid search approach. Values between 0 and 1 are examined by a small increment *l* = 0.01 up to *N*, where *N* is a large number chosen such that the value of the response time distribution at *N*λ is negligibly small for both decisions.

The recursive nature of the algorithm means that numerical precision errors accumulate as the sample being considered progresses. In practice, we found this sometimes necessitates a second corrective part to our numerical method. For later samples beyond a critical value, we fit the boundary a piece-wise linear curve, each segment containing 2–3 steps, minimizing the deviation between the simulated and the target first passage time distributions. The boundary that is found is thus a combination of the values returned by the algorithm up to the critical step, and brute-force piece-wise linear curve fitting.

## 3. Applications of our algorithm

In this section, we apply our algorithm to two problems. The first problem is theoretical, and involves the relationship between diffusion classes of sequential-sampling models. The second problem is empirical, and involves finding the time-varying boundaries for individual subjects from their behavioral data in key trials of a simple perceptual decision-making task.

### 3.1. Equating accumulator and diffusion models

**Figure 2:**
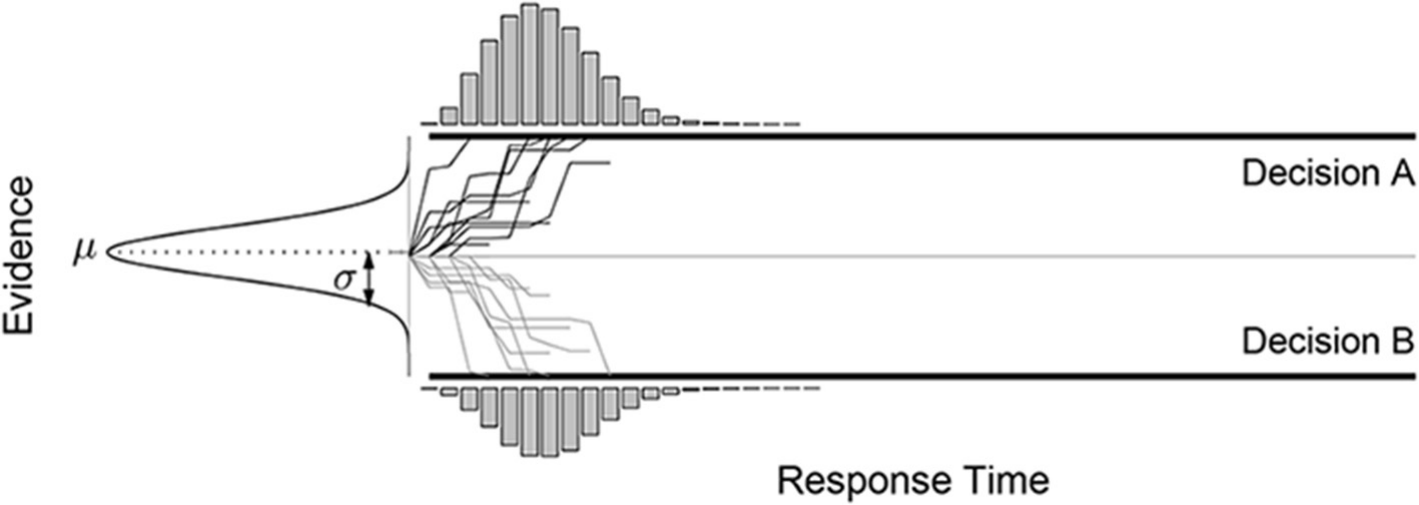
**The accumulator sequential sampling model**.

Within the sequential sampling framework, an alternative to the class of diffusion model is the class of *accumulator* models (Vickers, 1970, 1979). As shown in Figure 2, accumulator models maintain two separate evidence tallies, one for each alternative decision. Each sampled piece of evidence favors one or the other decision, and only those samples that favor a decision are added to their corresponding tally. The first tally to reach the boundary results in that decision being made, and the response time is the number of samples required for this to happen.

Because of their different evidence accrual mechanisms, diffusion and accumulator model are usually regarded as being qualitatively different, and treated as competing accounts of human decision making. Empirically, the standard conclusion is that diffusion models are superior accounts of data (e.g., Ratcliff and Smith, 2004), although there are some studies that find in favor of accumulator models (e.g., Lee and Corlett, 2003). Bogacz et al. (2006) compare diffusion and accumulator models theoretically, in terms of a set of optimality properties, and conclude that accumulator models cannot be reduced to diffusion models.

Complementing this focus on the two models as competing accounts of human decision-making, a natural application of our method is to find the time-varying boundaries that make a diffusion model *equivalent* to an accumulator model with constant boundaries and the same Gaussian evidence distribution. This goal can be seen as a natural extension of the long-standing equivalence result presented by Pike (1968) between random-walk and race models, which are the discrete analogs, respectively, of diffusion and accumulator models. Pike, (1968, Section 4.3) showed that, when the evidence samples are unit increments or decrements, simple time-varying boundaries, decreasing one unit in each time step, make the random-walk decisions and response-time distributions equivalent to the race model.

Formally, we consider the accumulator model sampling evidence from the Gaussian distribution with mean μ and standard deviation σ, and with a fixed starting point 0 and symmetric thresholds. This model generates a decision probability *p*^acc^ for choosing decision A, and response time distributions *r*^acc^_*A*_ and *r*^acc^_*B*_ for the two decisions. Thus, the accumulator model can be conceived as the mapping

(5) $$m^{\text{acc}}:(\mu ,\sigma )\to (p^{\text{acc}},r_{A}^{\text{acc}},r_{B}^{\text{acc}}).$$

Equating accumulator and diffusion models requires finding the boundaries *a_A_* (*n*) and *a_B_* (*n*), such that (*p*^acc^, *r*^acc^_*A*_, *r*^acc^_*B*_) = (*p*^diff^, *r*^diff^_*A*_, *r*^diff^_*B*_).

The mapping *m*^acc^ has been well-studied. Smith and Vickers (1988) provided an analytical expression, in the form of convolutions of the evidence distribution. For Gaussian evidence distributions, there is no closed-form solution, but a discrete approximation method is provided by Smith and Vickers (1989). In particular, we used the method detailed by Smith and Vickers, (1989, Appendix A). Their Equations A3a and A3b define *P_A,n_* and *P_B,n_* which are, respectively, the probability the accumulator model will choose alternative “A” or “B” after *n* samples.

**Figure 3:**
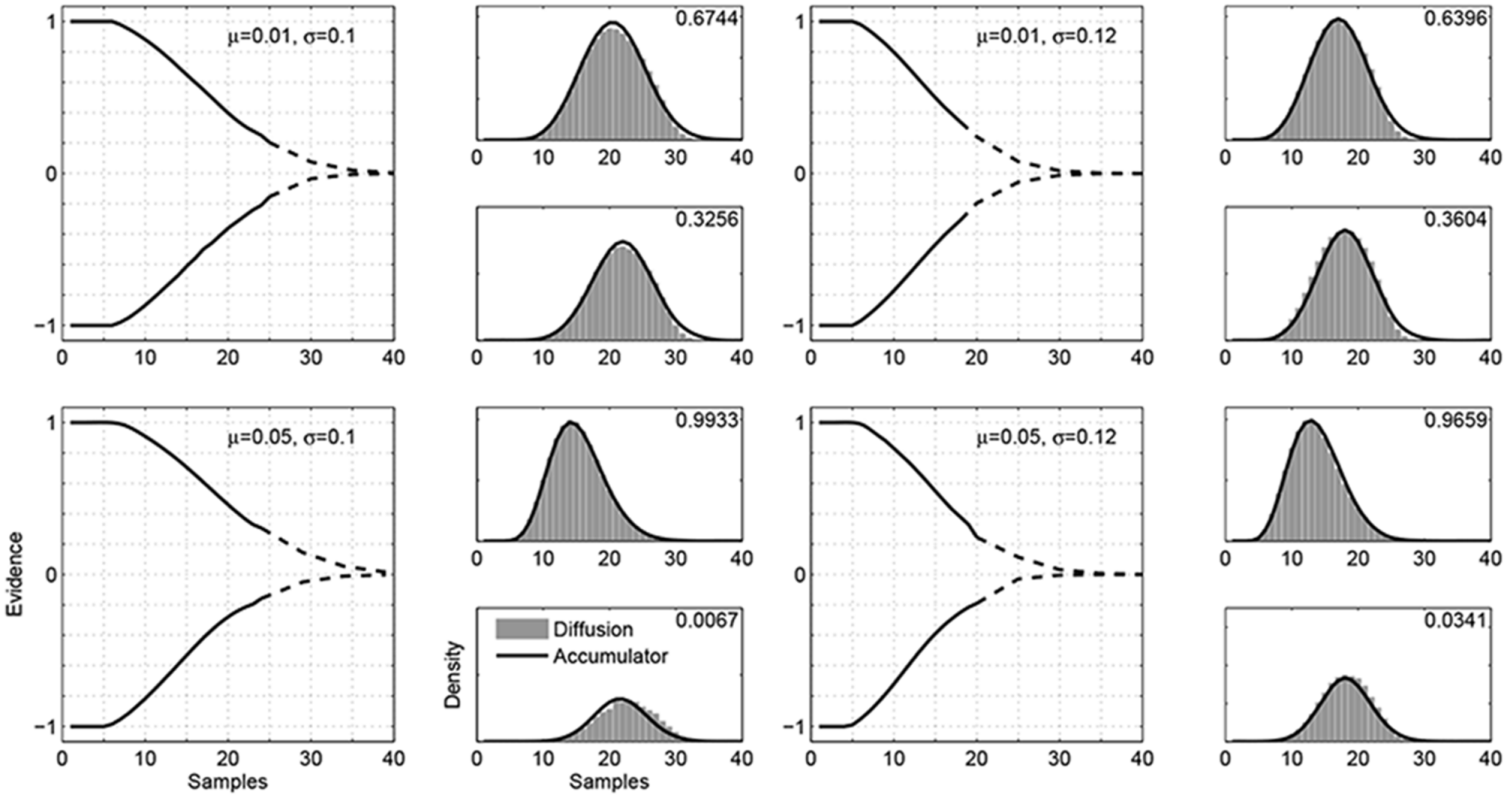
**Examples of the boundaries that equate a diffusion evidence accrual process with an accumulator model using constant boundaries**. The four examples correspond to four different evidence distributions, with values for the means μ and standard deviations σ indicated in the panels. In each example, the left-hand panel shows the time-varying boundaries found by our algorithm, with the part of the boundary found by the main algorithm shown as a solid line, while the part found by the piece-wise approximation shown as a broken line. The right-hand panels show the response time distributions for the two boundaries, weighted by the decision probability for each alternative. The accumulator distributions are shown as solid lines, and the diffusion distributions are shown by gray histograms. The values in the top-right corners show the choice probabilities.

Figure 3 shows four examples of the boundaries found by our algorithm. Each example corresponds to a different Gaussian evidence distribution, using means of μ = 0.01 and μ = 0.05 and standard deviations of σ = 0.1 and σ = 0.12. For these parameter combinations, we generated response-time distributions from an accumulator model. These distributions provided the input to our algorithm.

The boundaries found by the algorithm are shown in the main left-hand panel for each example in Figure 3. The part of the boundary found by the main algorithm is shown as a solid line, while the part found by the piece-wise approximation is shown as a broken line.^3^ The basic result is that the decision probabilities and response-time distributions generated by accumulator models correspond to those generated by a diffusion evidence accrual process with time-varying boundaries.

The right-hand panels in Figure 3 correspond to the two-decision alternatives, and show the accumulator and diffusion response-time distributions, as solid lines and gray histograms, respectively. These distributions are weighted by the decision probabilities, and so capture all of the aspects of model behavior that need to be equated. It is clear that the decision probabilities and response times generated by the diffusion evidence accrual process with the time-varying boundaries are very close to the target accumulator model distributions.

The four evidence distributions illustrated in Figure 3 span the interesting range of possibilities. They include cases where the response time distributions are skewed as well as symmetric, and cases where the mean response times for the two decisions are very different as well as very similar. They also include a wide range of decision probabilities, ranging from close to 50% down to about 1%.

The basic result is that diffusion models with time-varying boundaries, of the type shown in Figure 3, produce the same decisions and response time distributions as accumulator models with constant boundaries. An important aspect of this result is that the boundaries are established before any particular evidence sequence is encountered. The nature of the boundaries is not developed or changed as evidence is sampled within a trial. While establishing equivalence dynamically by adapting to current evidence is an interesting research problem in its own right (e.g., Hockley and Murdock, 1987), the current results establish a more general equivalence. They show what sorts of time-varying boundaries make the diffusion approach to evidence accrual the same as standard accumulator approaches.

**Figure 4:**
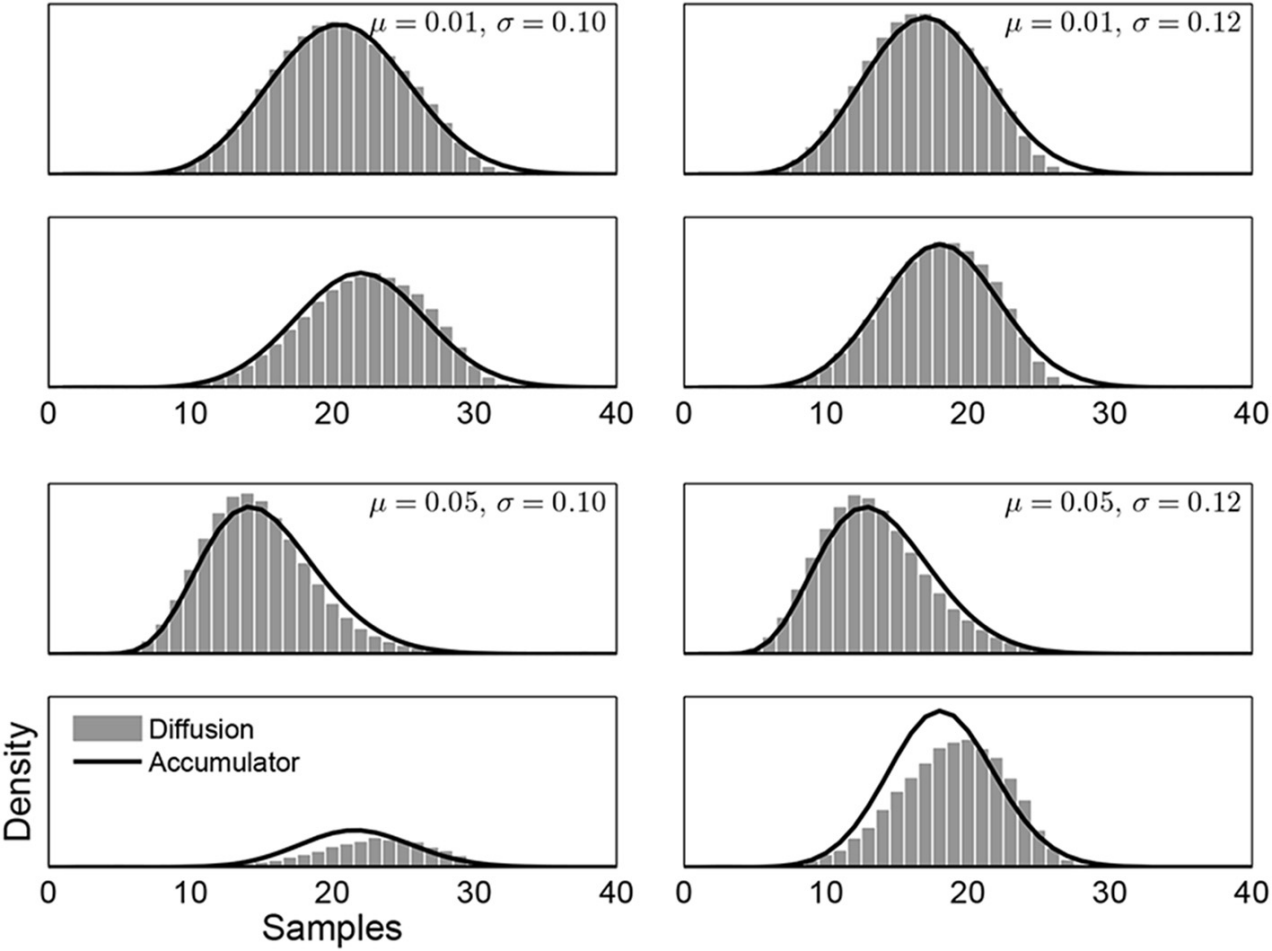
**Accumulator and diffusion response time distributions, under the constraint of symmetric boundaries, for four example evidence distributions**.

An interesting aspect of the results in Figure 3 is that it is clear that the time-varying boundaries are, in general, asymmetric. For example, when the evidence distribution is a Gaussian with μ = 0.05 and σ = 0.10, the lower boundary converges to zero more quickly than the upper boundary. Figure 4 presents a follow-up analysis, exploring how important symmetry is to equate accumulator and diffusion approaches to evidence accrual. Figure 4 shows the response-time distributions for the same examples considered in Figure 3, but using a modified algorithm that constrains the boundaries to be symmetric. For the evidence distributions with mean μ = 0.01 there is still close agreement between the accumulator and diffusion response time distributions. For the more extreme examples with mean μ = 0.05, the qualitative properties of different mean response times and negative skew are preserved, but there is quantitative disagreement between the accumulator and diffusion distributions.

### 3.2. Boundaries for ambiguous perceptual stimuli

One of the most intuitive motivations for considering diffusion models with time-varying boundaries relates to the case of non-evidential stimuli. These are stimuli that provide equal evidence for both response alternatives, and so the expectation of the evidence distribution is zero (i.e., μ = 0). For these stimuli, constant boundaries predict at least some extremely long response times, even though there is no information to be gained from repeated sampling from the stimulus. This prediction seems problematic, both empirically and theoretically, and has even led to sequential sampling models of human decision-making being lambasted in non-psychological literatures (Lamport, 2012). Converging boundaries provide a natural mechanism for ensuring a decision is made in a reasonable time, without needing to invoke additional psychological assumptions like over-riding termination processes.

Against this background, one interesting application of our method is to find the type of boundaries consistent with behavioral data for non-evidential stimuli. We consider data collected and analyzed by Ratcliff and Rouder (1998), which have also been examined by a number of other authors (e.g., Brown and Heathcote, 2005; Vandekerckhove et al., 2008). The Ratcliff and Rouder (1998) data involve three individual subjects each doing about 8000 trials over 11 days on a brightness discrimination task, under both speed and accuracy instructions. The stimuli consist of visual arrays of black and white dots, with the number of black and white dots controlling the evidence they provide for the choice alternatives bright and dark. Of the 33 different levels of brightness considered by Ratcliff and Rouder (1998), we focus on just those stimuli with equal numbers of black and white dots that (objectively) provide no evidence for either response alternative.

To apply our algorithm to these data, we had to make a number of simplifying assumptions. First, we assumed that the drift rate was zero, because of the objective properties of the stimuli. Obviously, it is possible that psychologically the stimuli are perceived as favoring one alternative or the other, through some form of bias. Secondly, we shifted the response time distributions according to the smallest response time observed for each individual in each condition. This is a simple empirical approach that probably only roughly approximates the underlying time to encode and respond that requires the shift. Finally, because our method proved unstable with respect to the multi-modalities inherent in binned characterizations of the data, we first fit a Weibull function to the response time distributions, and applied our algorithm to samples from these distributions.

**Figure 5:**
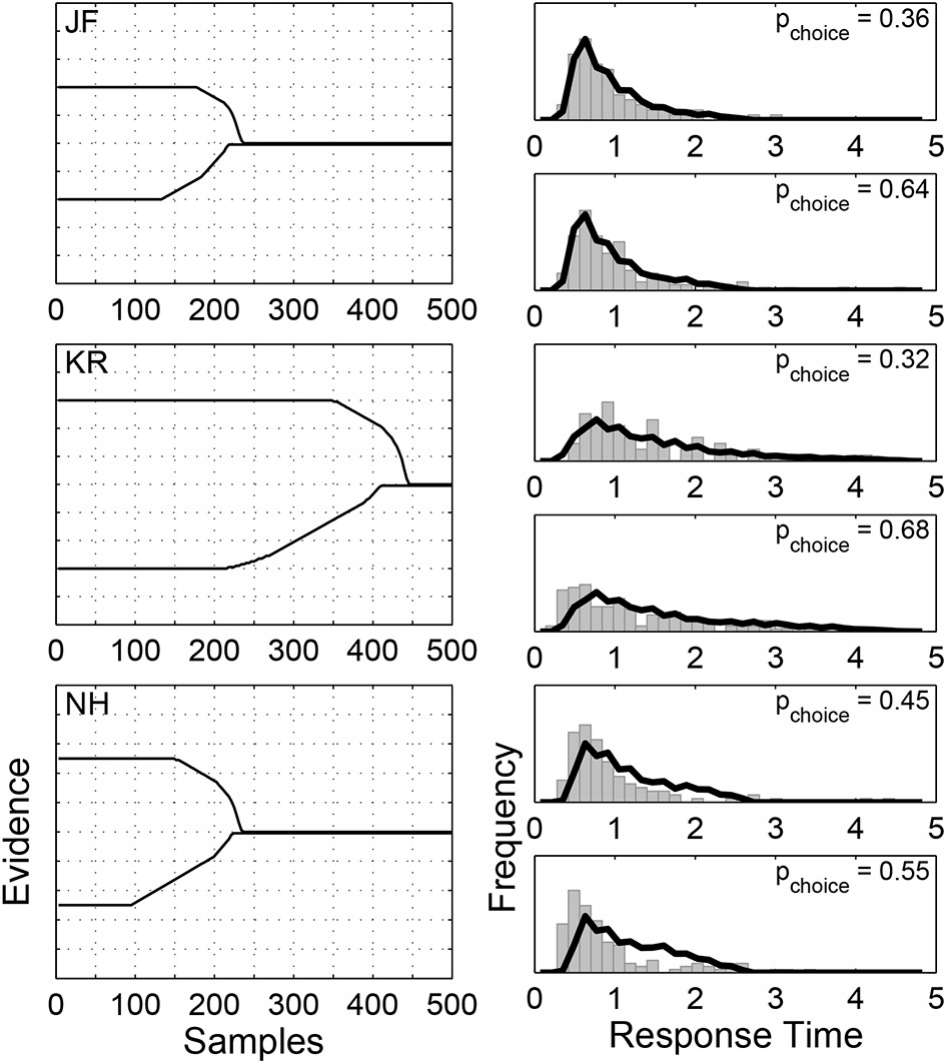
**Results of applying our algorithm to the three subjects from the Ratcliff and Rouder (1998) data, when viewing non-evidential perceptual stimuli in a brightness discrimination task under accuracy instructions**. The rows correspond to the three individual subjects:“JF,” “KR,” and “NH.” The main panels on the left show the boundaries found by our algorithm for the two decisions. The smaller panels on the right show the distribution of empirical response times (as gray histograms) and the distributions of response times generated by the boundaries (as solid lines) for the two decisions. The values in the top-right corners show the choice probabilities.

Figure 5 shows the results of our method on the Ratcliff and Rouder (1998) data, as applied to the accuracy condition.^4^ We used the Pearson's Chi-square tests standardly used in this literature^5^ to evaluate the goodness-of-fit of the Weibull distributions, binning the response times by decile, *d.f*. = 7. For subject “JF,” the Chi-square statistics and corresponding *p*-values for both alternatives are 7.14 (*p* = 0.41) and 13.02 (*p* = 0.07); for subject “KR,” they are 4.69 (*p* = 0.70) and 9.01 (*p* = 0.25); for “NH”, they are 10.02 (*p* = 0.19) and 10.99 (*p* = 0.14). The three rows in Figure 5 correspond to the three individual subjects:“JF,” “KR,” and “NH.” The main panels on the left show the boundaries found by our algorithm, with respond to discretized samples of 0.01 s duration. Here, we assume that every subject has the same evidence distribution, arbitrarily chosen to be *N*(0, 0.01), thus the starting values of the boundaries are now free parameters. The smaller panels on the right show the distributions of empirical response times (as gray histograms) and the distributions of response times generated by the time-varying boundaries found by our algorithm (as solid lines) for the two decision alternatives, measured in seconds. There is reasonably good agreement between these distributions, although it is better for some subjects (e.g., “JF”) than others. It is also clear that there are significant individual differences between the subjects, with “KR” taking longer to make decisions for these non-evidential stimuli.

Most interestingly, Figure 5 shows, once again, that the boundaries found are ones that converge asymmetrically. After an extended period of requiring the same level of evidence, both boundaries drop sharply toward zero and converge. They commence their descents at different times, though, with the lower boundary always converging first, but less sharply. Intuitively, when the stimulus favors neither alternative, symmetric boundaries should be able to fit the data well. We calculate the boundaries using the algorithm with the symmetry constraint in Appendix B, and find that the restricted algorithm finds boundaries close to the boundaries found by the original algorithm.

## 4. Discussion

Sequential sampling models are compelling accounts of the time course of human decision-making, based on the simple assumption that people sample information from a stimulus until they have enough evidence to make a decision. The default assumption in psychological modeling has been that the level of evidence required to make a decision does not change during this sampling process. The more general idea that the level of evidence might change during sampling is an appealing one, and the possibility that the evidence boundaries triggering decisions converge over time is an important one.

Most previous work dealing with time-varying boundaries has either involved assuming a parametric form for time-varying boundaries and fitting them to data (e.g., Milosavljevic et al., 2010; Ratcliff and Frank, 2012), fitting more general stochastic processes (e.g., Viviani, 1979), or making theoretical assumptions about optimality from which boundaries are derived by methods like dynamic programming (e.g., Frazier and Yu, 2008). In this paper, we have taken the first steps toward a more general approach that places minimal constraints on the form of time-varying boundaries, with the aim of finding their form from the response time distributions they produce.

We developed a method for finding time-varying boundaries that tries to solve the inverse problem of finding the boundaries that generate a given response time distribution for a known Gaussian evidence distribution. This method is related to current theoretical and practical work in statistics (e.g., Capocelli and Ricciardi, 1972; Cheng et al., 2006; Zucca and Sacerdote, 2009; Chen et al., 2011; Song and Zipkin, 2011). There remain important theoretical and practical gaps in these links, however, that future work should address. Theoretically, guarantees for the existence of time-varying boundaries being able to generate any response time distribution are available only for the single-boundary case. Practically, our current approach of solving an inverse problem can and should be generalized to one of solving an inference problem, placed priors on the time-varying boundaries that are possible, and expressing uncertainty over those possibilities based on available data. Our current algorithm, for example, does not allow for any characterization, such as a credible interval, of the uncertainty inherent in the fitted boundaries. Future work should aim to approach the problem as one of inference rather than inversion to provide this important information.

For these reasons, we think the two applications we presented of our method highlight the potential of the general approach, but constitute a starting point rather than a mature method. The theoretical application of our method showed that diffusion processes for accruing evidence, when allowed time-varying boundaries, produce the same behavior as the alternative class of accumulator accrual processes. This result is important, because it encourages a more general modeling perspective than seeing diffusion and accumulator models as incommensurable rivals. It also raises theoretical challenges, such as understanding the difference between what standard diffusion models with constant boundaries and standard accumulators are optimizing, and understanding the asymmetry of the boundaries that are inferred.

One interpretation of the asymmetry and its behavioral consequences is that accumulator evidence accrual is, in fact, fundamentally different from diffusion evidence accrual, in those situations where the decision-maker must be able to specify decision boundaries before a trial starts. This is because there is no way of knowing *a priori* which decision is favored by the stimulus, and so symmetry of the decision boundaries is a basic requirement. A counter-argument is that Figure 4 shows that imposing symmetry on the time-varying boundary still leads to close mimicry, and retains agreement on the fundamental qualitative features of the decisions and response times. Thus, it might be argued that there is a practical equivalence, in which empirical data might be equally well-explained by either model. In this sense, our analysis of the asymmetry raised more theoretical questions than it answered, but these questions would not have arisen or be able to be addressed without the capability to examine time-varying boundaries. Thus, we view this application of our method as one of those results that serves to sharpen the theoretical questions, and so usefully advances the field.

Similarly, our analysis of the response time distributions people produce when faced with perceptual stimuli that favored neither alternative is incomplete. We had to make a number of strong simplifying assumptions to apply our algorithm, and we think the boundaries we found should be treated as indicative rather than definitive. But this application did constitute a first productive step toward the important general goal of being able to find time-varying boundaries for diffusion models directly from individual-level behavioral data. The ultimate goal is an approach in which all of the relevant parameters, including properties of the evidence distribution, biases, encoding and responding times, and other properties of the decision-making process can be inferred simultaneously with unconstrained time-varying boundaries needed to account for a large set of empirical data varying across stimuli, task instructions, and other relevant manipulations.

Sequential sampling models are a powerful, popular, and important approach to understanding human decision-making. Extending these models to allow for time-varying boundaries has the potential to enhance greatly what they might help us learn about nature of human decision-making. We hope that the method developed and applied in this paper constitutes a first step toward realizing that potential.

### Conflict of interest statement

The authors declare that the research was conducted in the absence of any commercial or financial relationships that could be construed as a potential conflict of interest.

## Figures and Tables

**Figure A1:**
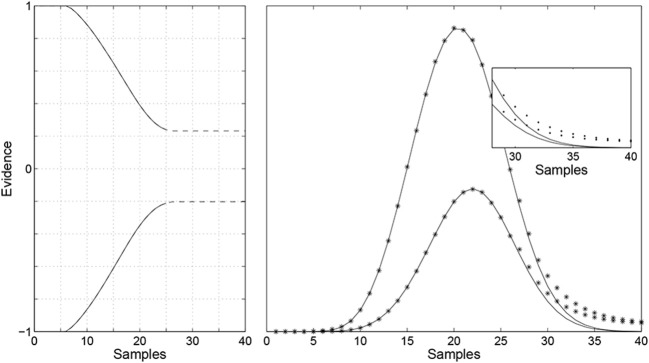
**Illustration of numerical problems with the basic algorithm The left-hand panel shows the boundary returned by the algorithm without correction**. The right-hand panel shows with asterisks the response time distributions generated by the boundaries in the left-hand panel. The target densities generated by the accumulator model are shown in solid lines.

**Figure A2:**
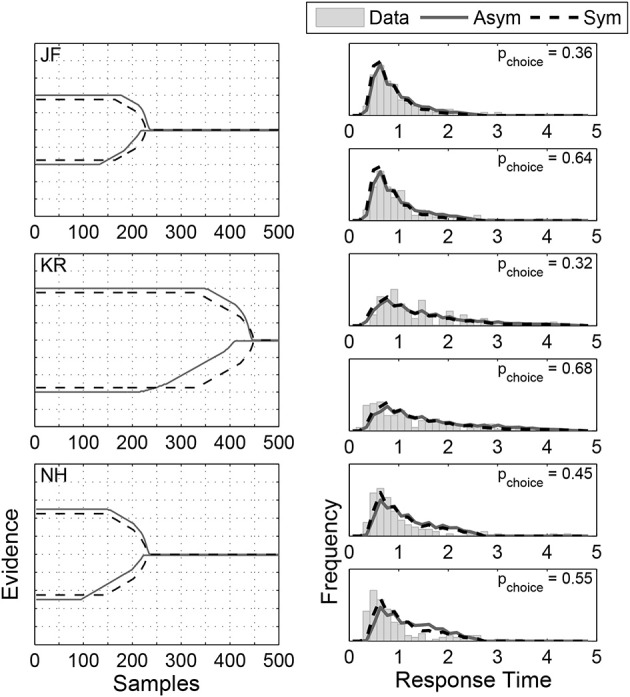
**Symmetric boundaries (shown as the broken lines) found for the three subjects using the restricted algorithm, superimposed with the asymmetric boundaries as in Figure 5**.

## Footnotes

^1^Simulations in Khodadadi et al. (2014), on the other hand, show that a Wiener process with constant boundaries is optimal for 2AFC with multiple difficulty levels, when a cue is added before each trial indicating the difficulty level of the upcoming trial.

^2^We use μ and σ to denote the mean and standard deviation of the evidence distribution, or *incremental distribution*, when we discretize the process to take samples from  (μ, σ). We use the standard notation ξ and s for the drift and the diffusion coefficient for the corresponding continuous drift diffusion process.

^3^The Appendix provides more detail on the piece-wise approximation in this application.

^4^We focused only on the accuracy condition, because we found the Weibull to be an inadequate characterization of the response time distributions in the speed condition.

^5^We are aware of the limitations of both the chi-square statistics and the use of *p*-values on which this analysis is based.

## Appendix A

Figure A1 shows the numerical problem in the main algorithm that requires the addition of the piece-wise linear correction. It shows the results of applying the unmodified Algorithm 1 to the response time distribution generated by an accumulator model with with Gaussian evidence distribution parameters μ = 0.01 and σ = 0.1 considered in the top-left of Figure 3. The left hand panel of Figure A1 shows the boundaries found, which differ from those in Figure 3 after the 26th sample, as indicated by the broken lines. The right hand panel of Figure A1 shows the target response time distributions generated by the accumulator model as solid lines, and the distributions generated from the boundary found by the unmodified algorithm as a line with asterisk markers. Using a small tolerance for the difference between these expected and generated distributions, it is possible to identify the critical point, highlighted by the magnification in the right hand panel, beyond which the piece-wise linear correction is applied.

## Appendix B

Figure A2 shows the results of applying a modified version of our algorithm that is constrained to find symmetric boundaries to the data from non-evidential stimuli for the three subjects considered by Ratcliff and Rouder (1998).
